# Radium-223 use and survival by line of treatment in metastatic castration-resistant prostate cancer: a nationwide population-based register study

**DOI:** 10.2340/1651-226X.2025.43794

**Published:** 2025-10-13

**Authors:** Charlotte Alverbratt, Fredrik Sandin, Viktor Kolmbäck, Hans Garmo, Ola Bratt, Ingela Franck Lissbrant

**Affiliations:** a Department of Oncology, Institute of Clinical Sciences, Sahlgrenska Academy, University of Gothenburg, Gothenburg, Sweden; b Department of Oncology, Halland Regional Hospital, Varberg, Sweden; c Regional Cancer Centre, Uppsala/Örebro, Uppsala University Hospital, Uppsala, Sweden; d Department of Surgical Sciences, Uppsala University, Uppsala, Sweden; e Department of Urology, Institute of Clinical Sciences, Sahlgrenska Academy, University of Gothenburg, Gothenburg, Sweden

**Keywords:** Prostatic neoplasms, castration-resistant, radium-223, observational study, neoplasm, metastasis, survival analysis

## Abstract

**Background:**

The role and optimal sequencing of radium-223 in the treatment of metastatic castration-resistant prostate cancer (mCRPC) remain debated. In Europe, radium-223 is restricted to third line treatment or later for chemotherapy-eligible men, although studies suggest greater benefit with earlier use. In this nationwide, population-based study, we investigated radium-223 use in Sweden and analyzed the association between line of treatment and overall survival.

**Methods:**

Men with mCRPC who started radium-223 in 2014-2020 were identified in national registers. The Kaplan-Meier method was used to estimate survival. The association between line of treatment and survival was analyzed with Cox regression and presented as adjusted hazard ratios (aHRs) with 95% confidence intervals (CIs). A subgroup with at least three mCRPC treatment lines was similarly analyzed.

**Results:**

1,133 men were included. Median overall survival was 13.9 months (95% CI 13.0-14.5). Later line of radium-223 treatment was associated with shorter survival; with first line as reference, aHR for death for second line was 1.34 (95% CI 1.12-1.59) and for third line 1.55 (1.29-1.87). The opposite was observed for 596 men with at least three lines of mCRPC treatment: aHR for second line was 0.80 (0.59-1.08) and for third line 0.78 (0.59-1.03).

**Interpretation:**

Survival after start of radium-223 in Sweden was comparable to pivotal trials, suggesting effective use. Our overall results do not suggest a better effect of radium-223 in first versus later mCRPC treatment lines but rather emphasize the value of a randomized controlled trial to more definitely determine the optimal timing of radium-223 treatment.

## Introduction

Radium-223, an alpha-emitter targeting bone metastases, was introduced in 2013 for treating metastatic castration-resistant prostate cancer (mCRPC) [1]. Radium-223 is now one of the many treatment options for this patient group. In addition to androgen receptor pathway inhibitors (ARPIs), chemotherapies, and combinations thereof in the hormone-sensitive phase, poly (ADP-ribose) polymerase (PARP)-inhibitors and prostate-specific membrane antigen (PSMA) targeting radioligand therapies have shown survival benefits. There is no consensus in international guidelines regarding the optimal sequencing of treatments for men with mCRPC, including radium-223 [2–5]. Ongoing trials are investigating how to optimize the use of radium-223 [6–8]. The PEACE-3 phase III trial study recently showed prolonged survival when adding radium-223 to enzalutamide [9].

With many treatments available, the choice of treatment in clinical practice depends on different guidelines, clinical practices, the physicians’ experiences, cancer characteristics, and patients’ preferences and comorbidities. The rapidly evolving landscape of mCRPC treatments makes it difficult to conduct randomized controlled trials (RCTs) to establish the optimal treatment sequencing. We must therefore rely on large observational studies with high-quality data to examine the effectiveness of treatments in real-world settings [10].

The pivotal ALSYMPCA trial demonstrated longer overall survival with radium-223 (median 14.9 vs placebo 11.3 months) in men with symptomatic mCRPC [1]. In a prespecified subgroup analysis, the survival benefit was independent of previous use of docetaxel [11]. Median survival from the start of radium-223 treatment varies greatly across observational studies, from 8 to 29 months, most of which included fewer than 300 men [12]. A large observational study reported that men surviving longer than 2 years after the start of radium-223 treatment had better performance status, lower disease burden, and less use of prior chemotherapy compared with those who survived less than 2 years [13].

In 2018, the European Medicines Agency (EMA) restricted the use of radium-223 to third or later line of treatment for chemotherapy-eligible patients because of an increased risk of bone fractures when combining radium-223 with abiraterone and prednisone [14, 15]. Regulatory agencies in the US, Canada, and Japan did not impose this restriction [4, 16]. The EMA decision to reserve radium-223 for later stages in the treatment pathway has been questioned, and earlier positioning advocated [2, 17–19] – not least because many studies have shown significant associations between earlier administration of radium-223 and improved survival, suggesting that this treatment is more effective in men with less advanced disease [18, 20–22]. One argument for earlier treatment is that radium-223 should be used before the disease has spread to visceral organs without radium-223 uptake [23, 24]. Restricting the use of radium-223 to later treatment lines may therefore result in missing the window of opportunity, potentially depriving men with mCRPC of a valuable treatment option [21, 25, 26].

In contrast to the abovementioned studies where authors argue for early use of radium-223 [18, 20–22], two observational studies have shown the opposite [27, 28]. Furthermore, a Swedish register study of fracture risk in men with mCRPC also reported overall survival stratified by their first line of treatment and found that men who started with radium-223 had shorter survival than the others [29].

Thus, guidelines for the use of radium-223 from regulatory authorities vary, and observational studies have reported conflicting results regarding line of treatment and survival. We therefore used nationwide population-based registers to investigate the use of radium-223 in Sweden and to try to establish the association between line of radium-223 treatment and overall survival for men with mCRPC while adjusting for several prognostic and clinical factors.

## Patients and methods

### Database

Prostate Cancer data Base Sweden (PCBaSe) is a database linking the National Prostate Cancer Register of Sweden (NPCR) with other healthcare registries by use of the unique Swedish person identity number [30]. NPCR captures 98% of all incident cases of prostate cancer in Sweden compared to the National Cancer Registry to which reporting is mandated by law. Registers linked in the PCBaSe include the Swedish National Cancer Registry, the National Patient Registry, the Cause of Death Registry, the Prescribed Drug Registry, and the Longitudinal Integrated Database for Health Insurance and Labour Market Studies.

PCBaSe also includes data from the Patient-Overview Prostate Cancer (PPC), a longitudinal register that, since 2014, provides clinicians with a graphical overview of information about the individual patient registered by the caregiver [31]. Data are prospectively collected during the patients’ clinical visits from diagnosis of prostate cancer to death, and we have previously shown high accuracy [32]. From 2014 until 2018, all information on radium-223 treatment was updated by a research nurse. During 2019–2020, the reporting clinics were responsible for updating the information. Research on data from PCBaSe is approved by the Swedish Ethical Review Authority (Reference number: 2022-05905-02), and this study was approved by a supplemental application.

### Study population

All men with mCRPC registered in PPC who received at least one injection of radium-223 outside clinical trials from 1st January 2014 to 31st December 2020 were included. Men with obviously inaccurate data in the database were excluded (i.e. date of drug stop before drug start). Follow-up ended on 9th February 2022.

### Variable definitions

Based on previous studies and clinical experience, we defined prognostic factors that may confound the analyses of line of treatment versus survival and categorized them as follows.

- **Variables reflecting aggressiveness of the disease at diagnosis** were primary treatment with or without curative intent (radiotherapy or radical prostatectomy) and time on androgen deprivation therapy (ADT) before castration-resistant disease, as a short time reflects a biologically more aggressive cancer [33]. The start of ADT was defined as the date for the first dispensed prescription of a GnRH agonist or an antiandrogen or of bilateral orchiectomy.
- **Disease-related variables at start of treatment** were serum prostatic-specific antigen (PSA), serum alkaline phosphatase (ALP), and blood hemoglobin (Hb) in the last 60 days before start of treatment (the latest value was used for the analyses if more than one value was available), regional lymph node metastases and visceral metastases, including lymph nodes outside the pelvis.
- **Variables related to the patient at start of treatment and his previous treatments** were age, Eastern Cooperative Oncology Group (ECOG) performance status, comorbidity, systemic treatment prior to CRPC, and pain. Comorbidity was assessed with Drug Comorbidity Index (DCI), Medical Drug Comorbidity Index (MDCI), and The Charlson Comorbidity Index (CCI). Systemic treatment (in addition to ADT) prior to CRPC included abiraterone/enzalutamide, docetaxel, the combination of them, and any other chemotherapy for prostate cancer. Pain was defined as a dispensed opioid prescription within 60 days before start of radium-223.

### Statistical methods

Survival was defined as time from the first injection of radium-223 to death or last date of follow-up. Overall survival was estimated with the Kaplan-Meier method, presented as curves and median times in months with 95% Confidence Intervals (CIs). Differences between survival curves were analyzed with the log rank test. The association between line of treatment and overall survival was analyzed with univariate and multivariate Cox regression models with adjustment for the potential confounders listed earlier, adding the groups one at a time. The results are presented as hazard ratios (HRs) with 95% CI. When adjusting for comorbidity, we used DCI and MDCI but not CCI, as CCI poorly predicts survival in men with CRPC [34–36].

To more specifically investigate whether the position of radium-223 in a treatment sequence is associated with survival, we analyzed a more homogeneous subgroup of men, all treated with at least three lines of therapies for mCRPC where radium-223 was given in first, second, or third line. Here, we defined survival as time from start of first line treatment (abiraterone/enzalutamide, docetaxel, or radium-223) to death or last date of follow-up. Overall survival and the association between the line of treatment for radium-223 and survival were analyzed and presented with the methods previously described for the whole cohort.

Missing data were estimated with multiple imputations by chained equation (MICE) with the assumption that they were missing at random. The imputation was replicated 20 times and stored in 20 different datasets. The variability between the imputed datasets was accounted for by Rubin´s rule for multiple imputations.

All data management and statistical analyses were conducted using R, version 4.2.2 (Vienna, Austria).

## Results

Of the 1,203 radium-223 treated men, 1,133 were eligible for the analysis, see flow chart in Supplemental Figure S1. Patient characteristics are displayed in Table 1. The median observation time from start of radium-223 to death or censoring was 13.9 months (IQR 7.8–24.5). At the end of the follow-up period, 1,045 men (92%) had died.

**Table 1:** Baseline characteristics at start of radium-223 treatment for the whole cohort (all patients in the study).

Characteristics	All		Line 1		Line 2		Line 3		Line ≥ 4	
	(*N* = 1,133)		(*N* = 293)		(*N* = 379)		(*N* = 335)		(*N* = 126)	
**Year of start of Ra-223 treatment (%)**										
2014	23	(2.0)	1	(0.3)	6	(1.6)	9	(2.7)	7	(5.6)
2015	212	(18.7)	56	(19.1)	48	(12.7)	68	(20.3)	40	(31.7)
2016	260	(22.9)	65	(22.2)	87	(23.0)	78	(23.3)	30	(23.8)
2017	305	(26.9)	117	(39.9)	92	(24.3)	78	(23.3)	18	(14.3)
2018	181	(16.0)	47	(16.0)	72	(19.0)	49	(14.6)	13	(10.3)
2019	67	(5.9)	4	(1.4)	35	(9.2)	21	(6.3)	7	(5.6)
2020	85	(7.5)	3	(1.0)	39	(10.3)	32	(9.6)	11	(8.7)
**Age, years**										
Median (IQR)	74	(69–79)	75	(70–80)	75	(70–80)	73	(69–77)	72	(68–75)
**Metastases at diagnosis (%)**										
Yes	457	(40.3)	135	(46.1)	159	(42.0)	117	(34.9)	46	(36.5)
No	676	(59.7)	158	(53.9)	220	(58.0)	218	(65.1)	80	(63.5)
**Curative treatment (%)**										
Yes	363	(32.0)	77	(26.3)	116	(30.6)	118	(35.2)	52	(41.3)
No	770	(68.0)	216	(73.7)	263	(69.4)	217	(64.8)	74	(58.7)
**Time from start of ADT to CRPC (%)**										
< 1 year	173	(15.3)	61	(20.8)	53	(14.0)	43	(12.8)	16	(12.7)
≥ 1 to < 3 years	450	(39.7)	113	(38.6)	153	(40.4)	128	(38.2)	56	(44.4)
≥ 3 to < 5 years	193	(17.0)	48	(16.4)	66	(17.4)	60	(17.9)	19	(15.1)
≥ 5 years	291	(25.7)	64	(21.8)	98	(25.9)	98	(29.3)	31	(24.6)
Missing	26	(2.3)	7	(2.4)	9	(2.4)	6	(1.8)	4	(3.2)
**ECOG (%)**										
0	390	(34.4)	126	(43.0)	113	(29.8)	112	(33.4)	39	(31.0)
1	472	(41.7)	106	(36.2)	165	(43.5)	142	(42.4)	59	(46.8)
≥ 2	169	(14.9)	37	(12.6)	69	(18.2)	50	(14.9)	13	(10.3)
Missing	102	(9.0)	24	(8.2)	32	(8.4)	31	(9.3)	15	(11.9)
**CCI (%)**										
0	435	(38.4)	122	(41.6)	147	(38.8)	120	(35.8)	46	(36.5)
1	130	(11.5)	31	(10.6)	46	(12.1)	46	(13.7)	7	(5.6)
2	145	(12.8)	43	(14.7)	53	(14.0)	33	(9.9)	16	(12.7)
≥ 3	423	(37.3)	97	(33.1)	133	(35.1)	136	(40.6)	57	(45.2)
**MDCI**										
Median (IQR)	1.4	(0.9–1.9)	1.3	(0.7–1.9)	1.4	(0.9–1.9)	1.4	(0.9–1.9)	1.6	(1.1–2.2)
**DCI**										
Median (IQR)	3	(1.8–4.4)	2.5	(1.3–4)	3.1	(1.8–4.5)	3.3	(1.9–4.6)	3.2	(2.1–4.6)
**Pain (%)**										
Yes	477	(42.1)	116	(39.6)	161	(42.5)	148	(44.2)	52	(41.3)
No	656	(57.9)	177	(60.4)	218	(57.5)	187	(55.8)	74	(58.7)
**Systemic treatment prior to CRPC (%)**										
Abiraterone/Enzalutamide	34	(3.0)	23	(7.8)	8	(2.1)	3	(0.9)	0	(0.0)
Taxane	147	(13.0)	45	(15.4)	54	(14.2)	42	(12.5)	6	(4.8)
Abiraterone/Enzalutamide + Taxane	17	(1.5)	10	(3.4)	4	(1.1)	2	(0.6)	1	(0.8)
Other CT	5	(0.4)	3	(1.0)	1	(0.3)	0	(0.0)	1	(0.8)
No treatment	930	(82.1)	212	(72.4)	312	(82.3)	288	(86.0)	118	(93.7)
**Systemic treatment prior to Ra-223 (%)**										
Abiraterone/Enzalutamide	321	(28.3)	0	(0.0)	269	(71.0)	52	(15.5)	0	(0.0)
Taxane	110	(9.7)	0	(0.0)	105	(27.7)	5	(1.5)	0	(0.0)
Abiraterone/Enzalutamide + Taxane	404	(35.7)	0	(0.0)	0	(0.0)	278	(83.0)	126	(100.0)
Other CT	5	(0.4)	0	(0.0)	5	(1.3)	0	(0.0)	0	(0.0)
No treatment	293	(25.9)	293	(100.0)	0	(0.0)	0	(0.0)	0	(0.0)
**Bone metastases (%)**										
Yes	1074	(94.8)	279	(95.2)	366	(96.6)	312	(93.1)	117	(92.9)
No	59	(5.2)	14	(4.8)	13	(3.4)	23	(6.9)	9	(7.1)
**Lymph node metastases (%)**										
Yes	348	(30.7)	70	(23.9)	113	(29.8)	117	(34.9)	48	(38.1)
No	785	(69.3)	223	(76.1)	266	(70.2)	218	(65.1)	78	(61.9)
**Visceral metastases (%)**										
Yes	82	(7.2)	11	(3.8)	29	(7.7)	26	(7.8)	16	(12.7)
No	1051	(92.8)	282	(96.2)	350	(92.3)	309	(92.2)	110	(87.3)
**Hemoglobin, g/l**										
Median (IQR)	126	(115.2–135)	127	(117–137)	126	(117–136)	126	(112.8–135.2)	122	(109–129.5)
Missing (%)	95	(8.4)	28	(9.6)	34	(9.0)	27	(8.1)	6	(4.8)
**PSA, ng/ml**										
Median (IQR)	94.7	(33–270)	55	(23–142.5)	86	(28–273)	119	(50.8–306.2)	163	(77.8–425)
Missing (%)	47	(4.1)	11	(3.8)	21	(5.5)	11	(3.3)	4	(3.2)
**ALP, µkat/l**										
Median (IQR)	2.5	(1.5–5.1)	2.7	(1.5–5.7)	2.4	(1.6–4.7)	2.4	(1.5–5.2)	2.3	(1.5–4.6)
Missing (%)	86	(7.6)	21	(7.2)	32	(8.4)	25	(7.5)	8	(6.3)
**Time first line treatment to death, months**										
Median (IQR)	28.3	(17.2–43)	15.8	(8.2–29.9)	26	(17.4–39.8)	33.9	(23.1–48.3)	45.5	(32.6–63.4)

Curative treatment: primary treatment with or without curative intent (radiotherapy or radical prostatectomy). ADT: androgen deprivation therapy; CRPC: castration-resistant prostate cancer; ECOG: Eastern Cooperative Oncology Group performance status; CCI: Charlson comorbidity index; MDCI: Medical Drug Comorbidity Index; DCI: Drug Comorbidity Index; pain: dispensed opioid prescription within 60 days before start of radium-223; systemic treatment prior to CRPC: abiraterone/enzalutamide, docetaxel, the combination of them, and any other chemotherapy for prostate cancer; lymph node metastases: regional lymph node metastases; visceral metastases: visceral metastases including lymph nodes outside the pelvis; PSA: serum prostatic-specific antigen, most recent registered value up to 60 days before treatment; ALP: serum alkaline phosphatase, most recent registered value up to 60 days before treatment.

Over half of the included men, 596 men received at least three lines of mCRPC treatment with radium-223, abiraterone/enzalutamide, and docetaxel and were included in the subgroup analysis. Their baseline characteristics are summarized in Table 2.

**Table 2:** Baseline characteristics at start of first line treatment for the subgroup (patients who received at least three lines of mCRPC therapy).

Characteristics	All		Ra-223 in Line 1		Ra-223 in Line 2		Ra-223 in Line 3	
	(*n* = 596)		(*n* = 82)		(*n* = 179)		(*n* = 335)	
**Year of start of first line treatment (%)**								
2008–2013	75	(12.6)	0	(0.0)	4	(2.2)	71	(21.2)
2014	103	(17.3)	1	(1.2)	22	(12.3)	80	(23.9)
2015	134	(22.5)	18	(22.0)	41	(22.9)	75	(22.4)
2016	120	(20.1)	20	(24.4)	61	(34.1)	39	(11.6)
2017	89	(14.9)	31	(37.8)	25	(14.0)	33	(9.9)
2018	46	(7.7)	9	(11.0)	13	(7.3)	24	(7.2)
2019	23	(3.9)	2	(2.4)	11	(6.1)	10	(3.0)
2020	6	(1.0)	1	(1.2)	2	(1.1)	3	(0.9)
**Age, years**								
Median (IQR)	71	(67–75)	71	(66.2–77)	72	(67–75)	71	(67–75)
**Metastases at diagnosis (%)**								
Yes	247	(41.4)	41	(50.0)	89	(49.7)	117	(34.9)
No	349	(58.6)	41	(50.0)	90	(50.3)	218	(65.1)
**Curative treatment (%)**								
Yes	201	(33.7)	21	(25.6)	62	(34.6)	118	(35.2)
No	395	(66.3)	61	(74.4)	117	(65.4)	217	(64.8)
**Time from start of ADT to CRPC (%)**								
<1 year	76	(12.8)	11	(13.4)	22	(12.3)	43	(12.8)
≥1 to <3 years	258	(43.3)	43	(52.4)	87	(48.6)	128	(38.2)
≥3 to <5 years	104	(17.4)	14	(17.1)	30	(16.8)	60	(17.9)
≥5 years	145	(24.3)	13	(15.9)	34	(19.0)	98	(29.3)
Missing	13	(2.2)	1	(1.2)	6	(3.4)	6	(1.8)
**ECOG (%)**								
0	349	(58.6)	47	(57.3)	102	(57.0)	200	(59.7)
1	157	(26.3)	27	(32.9)	47	(26.3)	83	(24.8)
≥2	24	(4.0)	3	(3.7)	11	(6.1)	10	(3.0)
Missing	66	(11.1)	5	(6.1)	19	(10.6)	42	(12.5)
**CCI (%)**								
0	297	(49.8)	43	(52.4)	92	(51.4)	162	(48.4)
1	86	(14.4)	9	(11.0)	28	(15.6)	49	(14.6)
2	59	(9.9)	5	(6.1)	18	(10.1)	36	(10.7)
≥3	154	(25.8)	25	(30.5)	41	(22.9)	88	(26.3)
**MDCI**								
Median (IQR)	1	(0.6–1.5)	1.1	(0.7–1.6)	1	(0.6–1.5)	1	(0.5–1.5)
**DCI**								
Median (IQR)	2.2	(1–3.6)	1.7	(0.9–3.6)	2	(0.7–3.5)	2.4	(1.3–3.8)
**Pain (%)**								
Yes	157	(26.3)	20	(24.4)	44	(24.6)	93	(27.8)
No	439	(73.7)	62	(75.6)	135	(75.4)	242	(72.2)
**Systemic treatment prior to CRPC (%)**								
Abiraterone/Enzalutamide	16	(2.7)	8	(9.8)	5	(2.8)	3	(0.9)
Taxane	88	(14.8)	20	(24.4)	26	(14.5)	42	(12.5)
Abiraterone/Enzalutamide + Taxane	4	(0.7)	1	(1.2)	1	(0.6)	2	(0.6)
Other CT	2	(0.3)	1	(1.2)	1	(0.6)	0	(0.0)
No treatment	486	(81.5)	52	(63.4)	146	(81.6)	288	(86.0)
**Bone metastases (%)**								
Yes	528	(88.6)	76	(92.7)	162	(90.5)	290	(86.6)
No	68	(11.4)	6	(7.3)	17	(9.5)	45	(13.4)
**Lymph node metastases (%)**								
Yes	173	(29.0)	20	(24.4)	52	(29.1)	101	(30.1)
No	423	(71.0)	62	(75.6)	127	(70.9)	234	(69.9)
**Visceral metastases (%)**								
Yes	27	(4.5)	4	(4.9)	11	(6.1)	12	(3.6)
No	569	(95.5)	78	(95.1)	168	(93.9)	323	(96.4)
**Hemoglobin, g/l**								
Median (IQR)	132	(123–140)	133	(122.5–141)	133	(123.8–141)	132	(123–140)
Missing (%)	154	(25.8)	3	(3.7)	59	(33.0)	92	(27.5)
**PSA, ng/ml**								
Median (IQR)	59	(24.3–165.2)	32.5	(13.5–92)	51	(18.2–139.8)	80.5	(31–183.8)
Missing (%)	22	(3.7)	0	(0.0)	5	(2.8)	17	(5.1)
**ALP, µkat/l**								
Median (IQR)	2.1	(1.4–3.8)	2	(1.3–3)	2	(1.4–3.4)	2.2	(1.4–4)
Missing (%)	118	(19.8)	4	(4.9)	45	(25.1)	69	(20.6)
**Time first line treatment to death, months**								
Median (IQR)	33.3	(22.3–46.4)	29.9	(18.7–38.9)	33.4	(23–45.8)	33.9	(23.1–48.3)
**Time first line treatment to third line treatment, months**								
Median (IQR)	19.3	(13.6–26.7)	16.1	(11.7–20.7)	19.5	(14.3–24)	20.6	(13.8–29.3)

Curative treatment: primary treatment with or without curative intent (radiotherapy or radical prostatectomy); ADT: androgen deprivation therapy; CRPC: castration-resistant prostate cancer; ECOG: Eastern Cooperative Oncology Group performance status; CCI: Charlson comorbidity index; MDCI: Medical Drug Comorbidity Index; DCI: Drug Comorbidity Index; pain: dispensed opioid prescription within 60 days before start of radium-223; systemic treatment prior to CRPC: abiraterone/enzalutamide, docetaxel, the combination of them, and any other chemotherapy for prostate cancer; lymph node metastases: regional lymph node metastases; visceral metastases: visceral metastases including lymph nodes outside the pelvis; PSA: serum prostatic-specific antigen, most recent registered value up to 60 days before treatment; ALP: serum alkaline phosphatase, most recent registered value up to 60 days before treatment.

**Figure 1:**
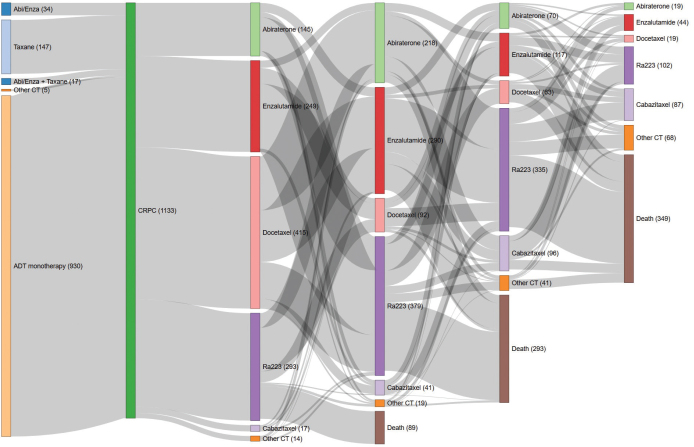
Line of sequence for radium-223-treated men with mCRPC in Sweden 2014–2020. Abi: Abiraterone; Enza: enzalutamide; Taxane: Docetaxel; CT: chemotherapy; ADT: androgen deprivation therapy; CRPC: castration-resistant prostate cancer; Ra-223: radium-223.

Radium-223 was mostly administered as second (33%) or third (30%) line mCRPC treatment. Men receiving radium-223 in the first line (26%) had less comorbidity and better performance status but more commonly had metastases at diagnosis, shorter time on ADT before CRPC, and less often received curative treatment at diagnosis. Thus, men receiving radium-223 in the first line were fitter at the start of treatment but had more aggressive prostate cancer at diagnosis than men receiving radium-223 in later treatment lines. Around one-third of men who received radium-223 as their first line treatment died before initiating any other treatments for CRPC (Figure 1). Less than 20% of all patients received docetaxel or abiraterone or the combination of both in the hormone-sensitive phase (Table 1).

Half of the men (51%) completed all the planned six cycles of radium-223 treatment. The completion rate of six cycles was higher when treatment was administered as the first line therapy (58%) compared with the fourth line or later therapy (44%). One quarter of all men discontinued treatment due to tumor progression, with a higher frequency observed when treated with radium-223 in the fourth line or later. Fewer than 10% terminated the radium-223 treatment due to side-effects (Table 3).

**Table 3:** Number of radium-223 cycles fulfilled and reason for treatment stop.

	All		Line 1		Line 2		Line 3		Line ≥ 4	
	(*N* = 1,133)		(*N* = 293)		(*N* = 379)		(*N* = 335)		(*N* = 126)	
**Number of radium-223 cycles (%)**										
1	51	(4.5)	6	(2.0)	22	(5.8)	15	(4.5)	8	(6.3)
2	81	(7.1)	14	(4.8)	29	(7.7)	27	(8.1)	11	(8.7)
3	128	(11.3)	31	(10.6)	55	(14.5)	31	(9.3)	11	(8.7)
4	98	(8.6)	22	(7.5)	31	(8.2)	25	(7.5)	20	(15.9)
5	99	(8.7)	29	(9.9)	28	(7.4)	32	(9.6)	10	(7.9)
6	576	(50.8)	171	(58.4)	187	(49.3)	163	(48.7)	55	(43.7)
≥ 7	1	(0.1)	0	(0.0)	0	(0.0)	1	(0.3)	0	(0.0)
Missing	99	(8.7)	20	(6.8)	27	(7.1)	41	(12.2)	11	(8.7)
**Reason for treatment stop (%)**										
According to plan	622	(54.9)	182	(62.1)	198	(52.2)	185	(55.2)	57	(45.2)
Toxicity	106	(9.4)	21	(7.2)	35	(9.2)	33	(9.9)	17	(13.5)
Progression	288	(25.4)	68	(23.2)	105	(27.7)	77	(23.0)	38	(30.2)
Patient’s choice	7	(0.6)	3	(1.0)	1	(0.3)	3	(0.9)	0	(0.0)
Death by progress	0	(0.0)	0	(0.0)	0	(0.0)	0	(0.0)	0	(0.0)
Death by toxicity	0	(0.0)	0	(0.0)	0	(0.0)	0	(0.0)	0	(0.0)
Death by other reason	7	(0.6)	3	(1.0)	2	(0.5)	1	(0.3)	1	(0.8)
Other reason	75	(6.6)	15	(5.1)	25	(6.6)	24	(7.2)	11	(8.7)
Missing	28	(2.5)	1	(0.3)	13	(3.4)	12	(3.6)	2	(1.6)

**Figure 2:**
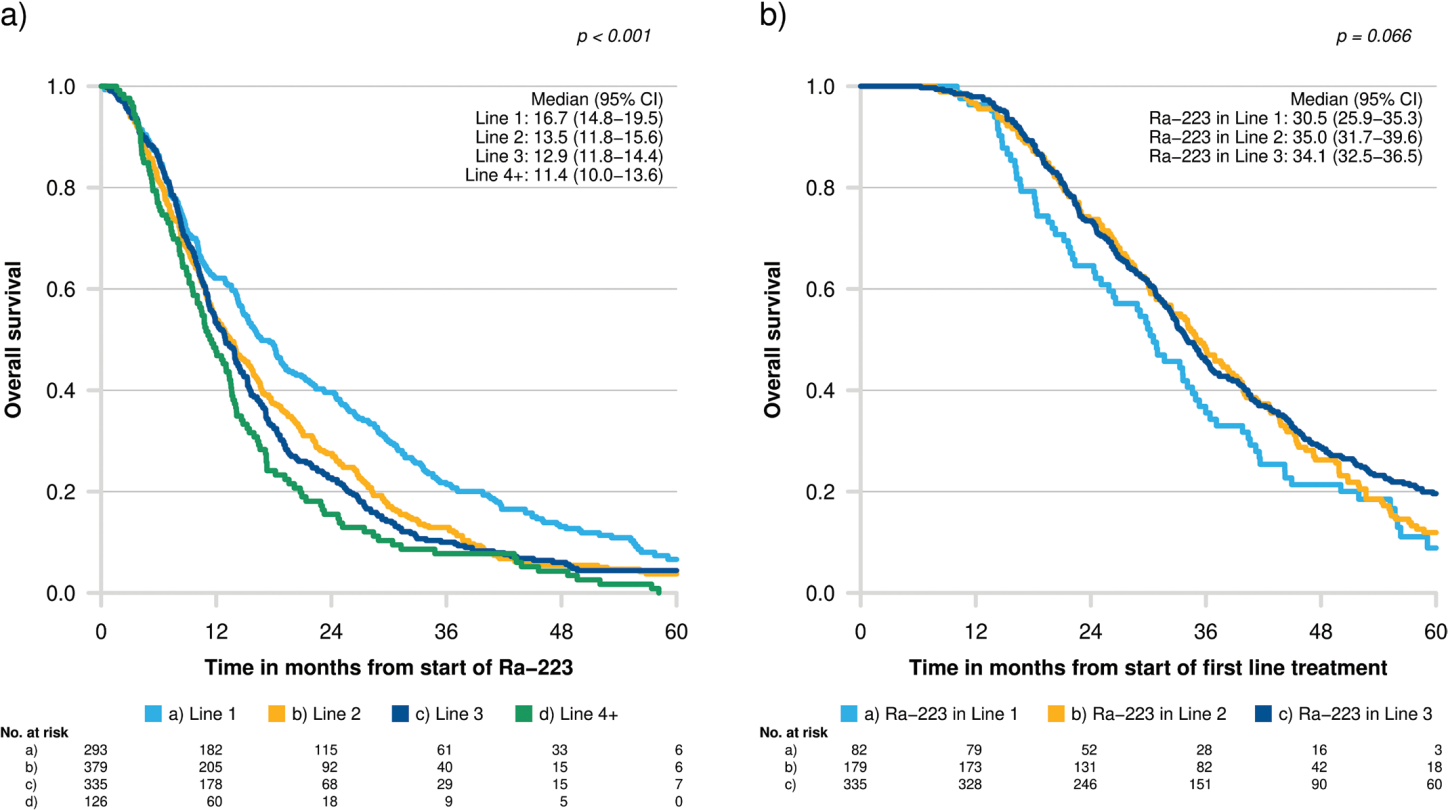
(a) Median (95% confidence interval) overall survival from start of radium-223 for the whole cohort; patients treated with radium-223 in lines 1, 2, 3, and 4 or later. (b) Median (95% confidence interval) overall survival from start of first line of mCRPC therapy for the subgroup: patients treated with at least three lines of treatment. mCRPC: metastatic castration-resistant prostate cancer; Ra-223: radium-223; No: number.

Median overall survival for the whole cohort was 13.9 (95% CI 13.0–14.5) months. Prostate cancer-specific survival was similar, 14.5 months (95% CI 13.9−15.5). Median overall survival was longer for men receiving radium-223 as the first line mCRPC treatment than for those receiving radium-223 later in the treatment pathway (*p* < 0.001), Figure 2a. However, among men who underwent at least three lines of mCRPC treatment, the opposite was found: men treated with radium-223 in the first line had shorter overall survival than men receiving it in the second or third line (*p* = 0.07), Figure 2b.

**Figure 3:**
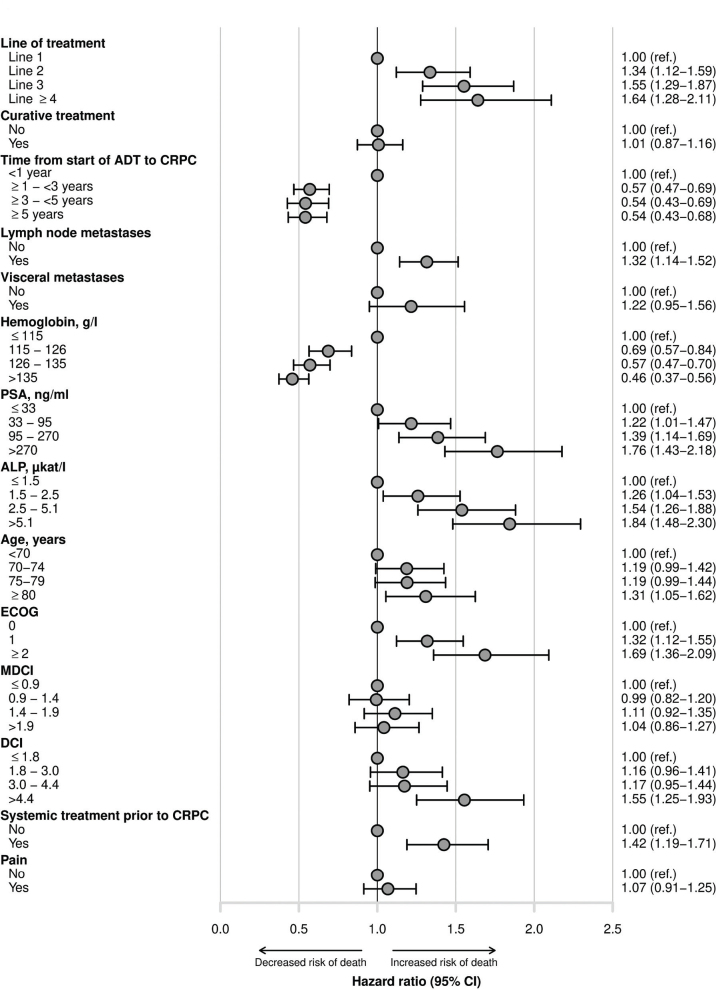
Forest plot of fully adjusted hazard ratios (HRs) with 95% confidence intervals (CIs), (uni- and multivariate analyses are presented in Supplemental Table S2) for the outcome death from any cause for prognostic factors at start of radium-223 treatment for the whole cohort. Line of treatment: line of radium-223 treatment in mCRPC setting; curative treatment: primary treatment with or without curative intent (radiotherapy or radical prostatectomy); ADT: androgen deprivation therapy; CRPC: castration-resistant prostate cancer; lymph node metastases: regional lymph node metastases; visceral metastases: visceral metastases including lymph nodes outside the pelvis; PSA: serum prostatic-specific antigen; ALP: serum alkaline phosphatase; Hb: Hemoglobin; ECOG: Eastern Cooperative Oncology Group performance status; MDCI: Medical Drug Comorbidity Index; DCI: Drug Comorbidity Index; systemic treatment prior to CRPC: abiraterone/enzalutamide, docetaxel, the combination of them, and any other chemotherapy for prostate cancer; pain: dispensed opioid prescription within 60 days before start of radium-223; CI: confidence interval.

Line of treatment remained significantly associated with survival in the whole cohort after full adjustment for potentially confounding variables, as shown in Figure 3. The HRs did not change much when more confounders were added in the multivariable analysis (Supplemental Table S2). The fully adjusted HRs for death were, compared with the use of radium-223 in the first line, 1.34 (95% CI 1.12–1.59) for use in the second line, 1.55 (1.29–1.87) in the third line, and 1.64 (1.28–2.11) in the fourth line or later. Other prognostic factors with significant HRs for death were short time on ADT, lymph node metastases, low Hb, high PSA and high ALP, poor ECOG performance status, and systemic treatment in addition to ADT prior to CRPC.

**Figure 4:**
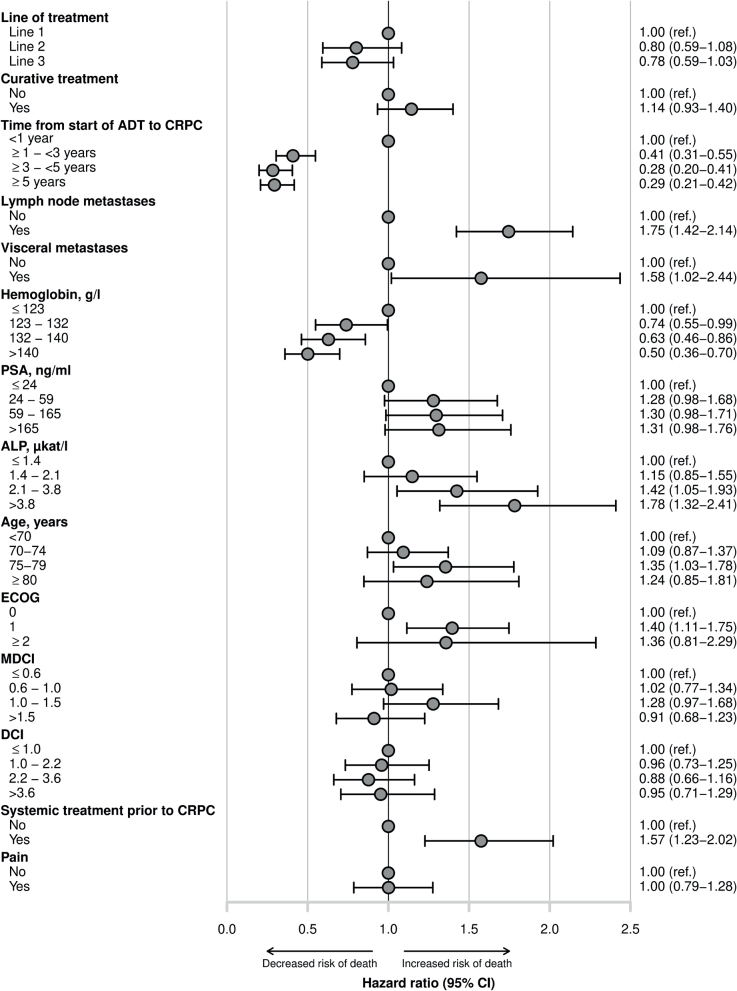
Forest plot of fully adjusted hazard ratios (HRs) with 95% confidence intervals (CIs), (uni- and multivariate analyses are presented in Suppemental Table S3) for the outcome death from any cause for prognostic factors at start of first line of mCRPC treatment for the subgroup: patients treated with at least three lines of treatment. Line of treatment: line of radium-223 treatment in mCRPC setting; curative treatment: primary treatment with or without curative intent (radiotherapy or radical prostatectomy); ADT: androgen deprivation therapy; CRPC: castration-resistant prostate cancer; lymph node metastases: regional lymph node metastases; visceral metastases: visceral metastases including lymph nodes outside the pelvis; PSA: serum prostatic-specific antigen; ALP: serum alkaline phosphatase; Hb: Hemoglobin; ECOG: Eastern Cooperative Oncology Group performance status; MDCI: Medical Drug Comorbidity Index; DCI: Drug Comorbidity Index; systemic treatment prior to CRPC: abiraterone/enzalutamide, docetaxel, the combination of them, and any other chemotherapy for prostate cancer; pain: dispensed opioid prescription within 60 days before start of radium-223; CI: confidence interval.

In contrast, for the subgroup with at least three lines of mCRPC treatment, the fully adjusted analysis favored late radium-223 treatment: the fully adjusted HRs for death were 0.80 (95% CI 0.59–1.08) for use in the second line and 0.78 (0.59–1.03) for use in the third line compared with use in the first line, Figure 4. Also in the subgroup, the multivariable analyses (Supplemental Table S3) show that the HRs did not change much when groups of confounding variables were adjusted for, and here, the fully adjusted result was not significant.

## Discussion and conclusions

In this long-term nationwide register-based Swedish study of radium-223 treated men with mCRPC, the median overall survival was similar as in the pivotal randomized trials and major observational studies, suggesting effective use. Most of the men were treated in second and third lines, harmonizing with today’s recommendations by the European Medicines Agency (EMA). Our two different analytical approaches to investigate the association between the line of treatment for radium-223 and survival showed notably discrepant results, so we could neither confirm nor refute the hypothesis that radium-223 is more effective when used in an early line of mCRPC treatment.

The main strengths of this study are its nationwide population-based design, and the access to extensive high-quality register data with life-long follow-up for over 90% of men. The high granularity of the register data allowed for adjustment for more confounders than in previous observational studies [20, 21]. A further strength is that we tested our hypothesis in both the whole cohort and in a more homogeneous subgroup to minimize residual confounding. Another notable strength is that we included data on all systemic treatments given before and after radium-223 treatment, not only in the CRPC setting but also in the hormone-sensitive phase of the prostate cancer. This approach provides a representative real-world evaluation of radium-223 use and effectiveness, which incorporated various new treatment options that were not available when radium-223 was introduced.

A weakness is some missing data, primarily related to laboratory results and performance status. This was managed with multiple imputations. Additionally, we lacked data on the number of bone metastases at start of radium-223 and on treatment response. It is, however, widely recognized that assessing response to radium-223 treatment is challenging [37]. Another possible weakness is that reporting of radium-223 treatments for 2019–2020 was not specifically updated by a research nurse in the Patient Overview Prostate Cancer (PPC) register, as happened during 2014–2018. Therefore, we cannot rule out the possibility of lower coverage in PPC during this period, but we do not believe that this has affected our outcome measures.

Median overall survival in our study was 13.9 months, essentially similar as in the phase-3 trial ALSYMPCA (14.9 months), even though men in our study were older and had more comorbidity at start of treatment, and over 40% of men in our study received radium-223 as a third line or later. In ALSYMPCA, all men received radium-223 in the first or second line.

Observational studies, including ours, have shown comparable results on overall survival. The EPIX study, a retrospective study of electronic health records in the US Flatiron database 2013–2019, presented a median overall survival of 12.9 months for radium-223-treated men and had a smaller proportion of men with performance status ECOG 0–1 in comparison to our cohort, 45% vs. 75% [13]. A large global prospective observational trial, REASSURE, showed overall survival of 15.6 months, but here, first line radium-223 treatment predominated [38]. A smaller nationwide Finish register study reported an overall survival of 13.8 months, with 25% of the men receiving radium-223 as the first line mCRPC treatment [39].

As shown in Figure 2a and in agreement with other observational studies [20, 21], men treated with radium-223 in later lines have a shorter average overall survival in comparison to men treated in the first line. This was expected, as men treated later in the mCRPC treatment pathway typically have more advanced disease and are in poorer health. Therefore, adjusted analysis is necessary to account for cancer and patient-related prognostic factors that may confound the association between the line of treatment and survival. In an exploratory analysis of the ALSYMPCA trial and in a phase 3b trial, prognostic factors for overall survival for radium-223-treated men were ECOG; laboratory values; PSA, ALP, Hb, and lactate dehydrogenase (LDH); and opiate use, age, and pain [40, 41]. An observational study by Kuppen and co-workers of 285 men showed significantly shorter overall survival with radium-223 in the third or later line compared with earlier treatment lines. The analysis adjusted for age, CCI, ECOG, lymph node involvement, visceral disease, and time from castration to mCRPC, Hb, ALP, LDH, and PSA, and the authors concluded that poorer survival was only partially explained by worse baseline characteristics at the start of radium-223 treatment [21]. Jarvis and co-workers studied 191 men and reported better survival for men treated in earlier lines compared with later lines, but they did not adjust for prognostic factors [20].

In our study, we adjusted for all prognostic factors in the trials mentioned earlier, except LDH, plus two other factors suggested by clinical experience: systemic treatment (in addition to ADT) prior to CRPC and primary treatment with curative intent. As shown in Figure 3, our findings confirm previously reported prognostic factors for survival among men treated with radium-223 [2]. We also found that systemic treatment (in addition to ADT) prior to the development of CRPC was negatively associated with survival. A likely explanation is that combination therapy for hormone-sensitive *de novo* metastatic disease reflects more aggressive disease. Still, after adjustments of all mentioned prognostic factors, first line treatment with radium-223 was associated with longer overall survival. Notably, in our study, the HRs changed remarkably little from the unadjusted to the adjusted analysis, suggesting considerable residual confounding. An important residual confounder was number of bone metastases, which is associated with overall survival [1, 42, 43]. Additionally, the physicians’ individualized treatment choices and patient´s preferences likely introduced selection bias that is difficult to adjust for, even when analyzing a relatively homogenous group.

To overcome some of the residual confounding, we analyzed a subgroup of men who all received a minimum of three lines of mCRPC treatment. Interestingly, the unadjusted analyses for this subgroup showed that radium-223 as first line treatment was associated with shorter survival. After adjustment, the HR was similar but no longer statistically significant. One would assume larger differences in HR after adjustment for the defined confounders in our study. For example, additional systemic treatment in the hormone-sensitive phase is primarily used for de novo metastatic disease, which often is more advanced or aggressive than metachronous metastatic disease, and may trigger treatment-specific resistance mechanisms. In the subgroup analysis, 37% of men treated with radium-223 in the first line had received additional systemic treatment in the hormone-sensitive phase compared with 18% of men treated in the second line and 14% treated in the third line.

We used two different analytical approaches that gave notably discrepant results: the analysis of the whole cohort suggested longer survival for men treated with radium-223 in the first line, whereas the subgroup analysis of men who received at least three mCRPC treatment lines suggested the opposite. These opposing results, with HRs for death of 1.6 and 0.8 respectively, imply that powerful confounding and selection mechanisms are at play when assessing the association between line of treatment and survival. A partial explanation for survival benefit for later line of radium-223 treatment in the subgroup analysis may be that men receiving radium-223 in second or third line could not have developed visceral metastases after their first line treatment (if they had, they would not have received radium-223 in later lines). This contrasts with men receiving radium-223 in the first line who could develop visceral metastases before initiating subsequent treatments, such as abiraterone/enzalutamide or docetaxel in later lines, and thereby had a higher risk of death.

In the ALSYMPCA trial, the recommended treatment consisted of six cycles of radium-223. The overall completion rate in our study (51%) was lower than in both the ALSYMPCA trial (63%) and the REASSURE trial (59%) but in line with the Dutch observational study by Kuppen and co-authors (51%) [21]. The rate of discontinuation of drug due to adverse events in the ALSYMPCA was 16%, a higher number than in our study (9%) and in the Dutch observational study (11%) [21]. Since patients in ALSYMPCA were part of a phase-3 clinical trial, adverse events were probably more thoroughly investigated and reported than in daily clinical work. It remains to be shown how to increase the proportion of patients completing radium-223 treatment. However, the findings in ALSYMPCA indicate that, despite only 63% of the patients completing six cycles of radium-223 treatment, there is a survival benefit. In contrast to several other previous studies [37, 44–49], we did not analyze the association between the number of cycles received and survival. Many of these studies reported longer survival in men receiving six cycles. However, none of them accounted for the immortal time bias introduced by the fact that men must be alive to receive all six cycles.

This large, nationwide register-based study with a long follow-up suggests effective use of radium-223 in Sweden in 2014-2020, with similar overall survival as in the pivotal randomized trials despite that the Swedish men were older and had more comorbidities. Our overall results do not confirm previous reports of a survival benefit for radium-223 in first versus later mCRPC treatment lines. These results emphasize the value of a randomized controlled trial to more definitively determine the optimal timing of radium-223 administration in this patient population.

## Supplementary Material



## Data Availability

Data used for the current study have been extracted from the Patient-overview Prostate Cancer (PPC), part of the National Prostate Cancer Register (NPCR) of Sweden. These data can be retrieved after application to the steering groups of NPCR. For more information, please see www.npcr.se/in-english where registration forms, manuals, and annual reports from NPCR are found as well as a full list of publications from PCBaSe. Email address: contact npcr@npcr.se.
