# Supplementary material for: Radium-223 use and survival by line of treatment in metastatic castration-resistant prostate cancer: a nationwide population-based register study
Source: Acta Oncol. 2025 Oct 13;64:43794. doi: 10.2340/1651-226X.2025.43794 (PMC12535217; doi:10.2340/1651-226X.2025.43794)
Supplement: Supplementary file 1 [file AO-64-43794-s1.pdf]

Supplementary material has been published as submitted. It has not been copyedited, or typeset by Acta Oncologica

Supplemental Material

Supplemental Figure S1:

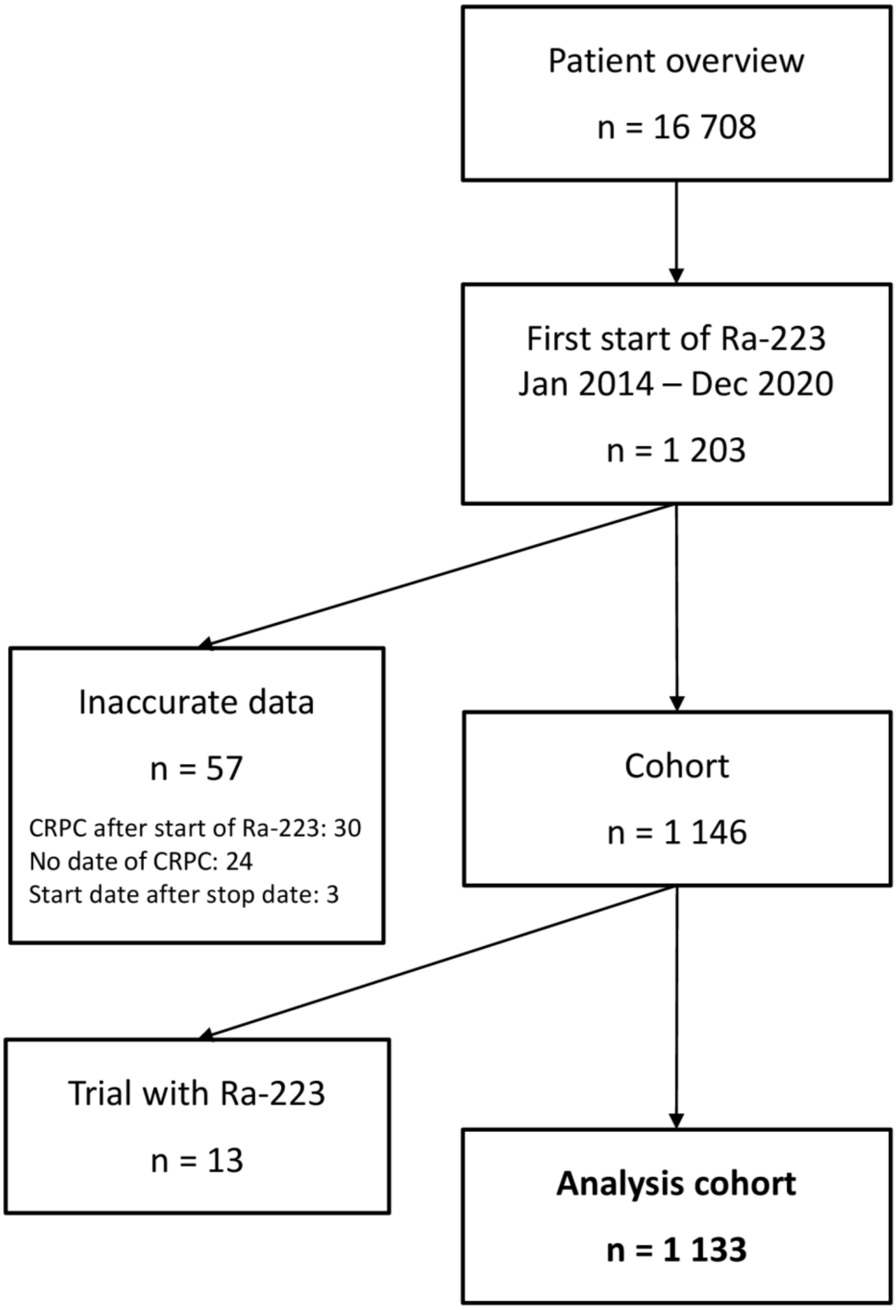

Supplemental Table S2:	Univariate			Multivariate Step 1			Multivariate Step 2			Multivariate Step 3		
	HR	(95% CI)	*p-value*	HR	(95% CI)	*p-value*	HR	(95% CI)	*p-value*	HR	(95% CI)	*p-value*
**Line of treatment**												
Line 1	1.00	(ref.)		1.00	(ref.)		1.00	(ref.)		1.00	(ref.)	
Line 2	1.34	(1.14-1.57)	*<0.001*	1.39	(1.19-1.64)	*<0.001*	1.42	(1.20-1.69)	*<0.001*	1.34	(1.12-1.59)	*0.001*
Line 3	1.41	(1.20-1.67)	*<0.001*	1.50	(1.27-1.77)	*<0.001*	1.55	(1.30-1.86)	*<0.001*	1.55	(1.29-1.87)	*<0.001*
Line ≥4	1.75	(1.41-2.17)	*<0.001*	1.83	(1.47-2.27)	*<0.001*	1.59	(1.25-2.01)	*<0.001*	1.64	(1.28-2.11)	*<0.001*
**Curative treatment**												
No	1.00	(ref.)		1.00	(ref.)		1.00	(ref.)		1.00	(ref.)	
Yes	0.88	(0.77-1.00)	*0.047*	0.88	(0.77-1.00)	*0.055*	0.95	(0.82-1.09)	*0.443*	1.01	(0.87-1.16)	*0.923*
**Time from start of ADT to CRPC**												
<1 year	1.00	(ref.)		1.00	(ref.)		1.00	(ref.)		1.00	(ref.)	
≥1 - <3 years	0.58	(0.48-0.70)	*<0.001*	0.55	(0.46-0.66)	*<0.001*	0.59	(0.49-0.72)	*<0.001*	0.57	(0.47-0.69)	*<0.001*
≥3 - <5 years	0.54	(0.44-0.67)	*<0.001*	0.53	(0.43-0.66)	*<0.001*	0.55	(0.44-0.69)	*<0.001*	0.54	(0.43-0.69)	*<0.001*
≥5 years	0.57	(0.47-0.69)	*<0.001*	0.55	(0.45-0.67)	*<0.001*	0.58	(0.47-0.71)	*<0.001*	0.54	(0.43-0.68)	*<0.001*
**Lymph node metastases**												
No	1.00	(ref.)					1.00	(ref.)		1.00	(ref.)	
Yes	1.35	(1.19-1.54)	*<0.001*				1.30	(1.13-1.49)	*<0.001*	1.32	(1.14-1.52)	*<0.001*
**Visceral metastases**												
No	1.00	(ref.)					1.00	(ref.)		1.00	(ref.)	
Yes	1.47	(1.17-1.84)	*0.001*				1.31	(1.03-1.68)	*0.029*	1.22	(0.95-1.56)	*0.122*
**Hemoglobin, g/l**												
Q1	1.00	(ref.)					1.00	(ref.)		1.00	(ref.)	
Q2	0.59	(0.49-0.70)	*<0.001*				0.66	(0.54-0.80)	*<0.001*	0.69	(0.57-0.84)	*<0.001*
Q3	0.44	(0.36-0.52)	*<0.001*				0.52	(0.42-0.63)	*<0.001*	0.57	(0.47-0.70)	*<0.001*
Q4	0.32	(0.27-0.38)	*<0.001*				0.39	(0.32-0.48)	*<0.001*	0.46	(0.37-0.56)	*<0.001*
**PSA, ng/ml**												
Q1	1.00	(ref.)					1.00	(ref.)		1.00	(ref.)	
Q2	1.37	(1.14-1.64)	*<0.001*				1.18	(0.98-1.42)	*0.083*	1.22	(1.01-1.47)	*0.042*
Q3	1.80	(1.50-2.16)	*<0.001*				1.43	(1.18-1.73)	*<0.001*	1.39	(1.14-1.69)	*0.001*
Q4	2.59	(2.14-3.14)	*<0.001*				1.78	(1.44-2.20)	*<0.001*	1.76	(1.43-2.18)	*<0.001*
**ALP, µkat/l**												
Q1	1.00	(ref.)					1.00	(ref.)		1.00	(ref.)	
Q2	1.29	(1.07-1.55)	*0.007*				1.27	(1.05-1.54)	*0.013*	1.26	(1.04-1.53)	*0.020*
Q3	1.84	(1.53-2.20)	*<0.001*				1.57	(1.29-1.91)	*<0.001*	1.54	(1.26-1.88)	*<0.001*

Q4	2.84	(2.35-3.42)	*<0.001*	2.04	(1.65-2.52)	*<0.001*	1.84	(1.48-2.30)	*<0.001*
**Age, years**									
<70	1.00	(ref.)					1.00	(ref.)	
70-74	1.14	(0.97-1.35)	*0.116*				1.19	(0.99-1.42)	*0.065*
75-79	1.09	(0.92-1.30)	*0.293*				1.19	(0.99-1.44)	*0.070*
≥80	1.28	(1.07-1.53)	*0.006*				1.31	(1.05-1.62)	*0.015*
**ECOG**									
0	1.00	(ref.)					1.00	(ref.)	
1	1.59	(1.38-1.83)	*<0.001*				1.32	(1.12-1.55)	*<0.001*
2+	2.19	(1.82-2.63)	*<0.001*				1.69	(1.36-2.09)	*<0.001*
**MDCI**									
Q1	1.00	(ref.)					1.00	(ref.)	
Q2	1.09	(0.92-1.30)	*0.316*				0.99	(0.82-1.20)	*0.947*
Q3	1.30	(1.09-1.55)	*0.003*				1.11	(0.92-1.35)	*0.282*
Q4	1.48	(1.25-1.76)	*<0.001*				1.04	(0.86-1.27)	*0.678*
**DCI**									
Q1	1.00	(ref.)					1.00	(ref.)	
Q2	1.38	(1.16-1.65)	*<0.001*				1.16	(0.96-1.41)	*0.131*
Q3	1.68	(1.41-2.00)	*<0.001*				1.17	(0.95-1.44)	*0.134*
Q4	2.40	(2.02-2.86)	*<0.001*				1.55	(1.25-1.93)	*<0.001*
**Systemic treatment prior to CRPC**									
No	1.00	(ref.)					1.00	(ref.)	
Yes	1.15	(0.98-1.35)	*0.078*				1.42	(1.19-1.71)	*<0.001*
**Pain**									
No	1.00	(ref.)					1.00	(ref.)	
Yes	1.62	(1.44-1.84)	*<0.001*				1.07	(0.91-1.25)	*0.410*

Supple-mental Table S3:	Univariate			Multivariate Step 1			Multivariate Step 2			Multivariate Step 3		
	**HR**	**(95% CI)**	***p-value***	**HR**	**(95% CI)**	***p-value***	**HR**	**(95% CI)**	***p-value***	**HR**	**(95% CI)**	***p-value***
**Line of Ra-223 treatment**												
Line 1	1.00	(ref.)		1.00	(ref.)		1.00	(ref.)		1.00	(ref.)	
Line 2	0.79	(0.60-1.05)	*0.101*	0.84	(0.64-1.12)	*0.236*	0.75	(0.56-1.01)	*0.057*	0.80	(0.59-1.08)	*0.150*
Line 3	0.74	(0.57-0.95)	*0.020*	0.80	(0.62-1.04)	*0.099*	0.71	(0.54-0.93)	*0.012*	0.78	(0.59-1.03)	*0.083*
**Curative treatment**												
No	1.00	(ref.)		1.00	(ref.)		1.00	(ref.)		1.00	(ref.)	
Yes	0.93	(0.78-1.11)	0.427	1.00	(0.84-1.21)	*0.973*	1.10	(0.90-1.33)	*0.363*	1.14	(0.93-1.40)	*0.198*
**Time from start of ADT to CRPC**												
<1 year	1.00	(ref.)		1.00	(ref.)		1.00	(ref.)		1.00	(ref.)	
≥1 - <3 years	0.47	(0.36-0.61)	<0.001	0.47	(0.36-0.62)	*<0.001*	0.45	(0.33-0.60)	*<0.001*	0.41	(0.31-0.55)	*<0.001*
≥3 - <5 years	0.36	(0.26-0.49)	<0.001	0.37	(0.27-0.50)	*<0.001*	0.31	(0.22-0.44)	*<0.001*	0.28	(0.20-0.41)	*<0.001*
≥5 years	0.36	(0.27-0.48)	<0.001	0.37	(0.27-0.50)	*<0.001*	0.32	(0.23-0.44)	*<0.001*	0.29	(0.21-0.42)	*<0.001*
**Lymph node metastases**												
No	1.00	(ref.)					1.00	(ref.)		1.00	(ref.)	
Yes	1.56	(1.30-1.88)	<0.001				1.74	(1.42-2.12)	*<0.001*	1.75	(1.42-2.14)	*<0.001*
**Visceral metastases**												
No	1.00	(ref.)					1.00	(ref.)		1.00	(ref.)	
Yes	1.53	(1.04-2.26)	0.031				1.48	(0.96-2.27)	*0.075*	1.58	(1.02-2.44)	*0.042*
**Hemoglobin, g/l**												
Q1	1.00	(ref.)					1.00	(ref.)		1.00	(ref.)	
Q2	0.68	(0.52-0.89)	0.005				0.71	(0.53-0.94)	*0.018*	0.74	(0.55-0.99)	*0.047*
Q3	0.63	(0.48-0.82)	<0.001				0.61	(0.45-0.82)	*0.001*	0.63	(0.46-0.86)	*0.004*
Q4	0.49	(0.38-0.64)	<0.001				0.49	(0.36-0.67)	*<0.001*	0.50	(0.36-0.70)	*<0.001*
**PSA, ng/ml**												
Q1	1.00	(ref.)					1.00	(ref.)		1.00	(ref.)	
Q2	1.18	(0.92-1.51)	0.195				1.28	(0.98-1.67)	*0.066*	1.28	(0.98-1.68)	*0.074*
Q3	1.09	(0.85-1.40)	0.473				1.24	(0.95-1.63)	*0.119*	1.30	(0.98-1.71)	*0.065*
Q4	1.25	(0.97-1.60)	0.089				1.31	(0.98-1.77)	*0.070*	1.31	(0.98-1.76)	*0.069*
**ALP, µkat/l**												
Q1	1.00	(ref.)					1.00	(ref.)		1.00	(ref.)	
Q2	1.09	(0.83-1.41)	0.546				1.11	(0.83-1.49)	*0.468*	1.15	(0.85-1.55)	*0.369*
Q3	1.23	(0.93-1.63)	0.140				1.38	(1.03-1.85)	*0.031*	1.42	(1.05-1.93)	*0.023*
Q4	1.85	(1.39-2.46)	<0.001				1.76	(1.29-2.38)	*<0.001*	1.78	(1.32-2.41)	*<0.001*
**Age, years**												
<70	1.00	(ref.)								1.00	(ref.)	
70-74	1.02	(0.83-1.25)	0.830							1.09	(0.87-1.37)	*0.446*

75-79	1.13	(0.90-1.41)	0.304	1.35	(1.03-1.78)	*0.029*
≥80	1.01	(0.72-1.40)	0.973	1.24	(0.85-1.81)	*0.268*
**ECOG**						
0	1.00	(ref.)		1.00	(ref.)	
1	1.59	(1.31-1.93)	<0.001	1.40	(1.11-1.75)	*0.004*
2+	1.58	(1.00-2.48)	0.051	1.36	(0.81-2.29)	*0.253*
**MDCI**						
Q1	1.00	(ref.)		1.00	(ref.)	
Q2	1.11	(0.87-1.42)	0.387	1.02	(0.77-1.34)	*0.901*
Q3	1.28	(1.00-1.62)	0.048	1.28	(0.97-1.68)	*0.081*
Q4	1.12	(0.88-1.43)	0.349	0.91	(0.68-1.23)	*0.538*
**DCI**						
Q1	1.00	(ref.)		1.00	(ref.)	
Q2	1.13	(0.89-1.44)	0.321	0.96	(0.73-1.25)	*0.756*
Q3	1.12	(0.88-1.42)	0.367	0.88	(0.66-1.16)	*0.361*
Q4	1.30	(1.02-1.65)	0.032	0.95	(0.71-1.29)	*0.752*
**Systemic treatment prior to CRPC**						
No	1.00	(ref.)		1.00	(ref.)	
Yes	1.53	(1.23-1.90)	<0.001	1.57	(1.23-2.02)	*<0.001*
**Pain**						
No	1.00	(ref.)		1.00	(ref.)	
Yes	1.31	(1.08-1.59)	0.006	1.00	(0.79-1.28)	*0.992*

Legends Supplement material

Suppl. Fig. S1: Flow diagram for selection of men included in the study.

Ra-223: radium-223, Jan: January, Dec: December, CRPC: castration resistant prostate cancer

Suppl. Table S2: Hazard ratios (HR) with 95% confidence intervals (CI) for death from any cause after start of radium-223 treatment for the whole cohort, adjustment with prognostic factors grouped; step 1: curative treatment, time from start of ADT to CRPC; step 2: lymph node metastases, visceral metastases, laboratory values of PSA, ALP, Hb; step 3: age, ECOG performance status, MDCI, DCI, systemic treatment prior to CRPC, pain.

Line of treatment: line of radium-223 treatment in mCPRC setting; curative treatment: primary treatment with our without curative intent (radiotherapy or radical prostatectomy); ADT: androgen deprivation therapy; CRPC: castration resistant prostate cancer; lymph node metastases: regional lymph node metastases; visceral metastases: visceral metastases including lymph nodes outside the pelvis; PSA: serum prostatic-

specific antigen; ALP: serum alkaline phosphatase; Hb: Haemoglobin; ECOG: Eastern Cooperative Oncology Group performance status; MDCI: Medical Drug Comorbidity Index; DCI: Drug Comorbidity Index; systemic treatment prior to CRPC: abiraterone/enzalutamide, docetaxel, the combination of them and any other chemotherapy for prostate cancer; pain: dispensed opioid prescription within 60 days before start of radium-223.

Suppl. Table S3. Hazard ratios (HR) with 95% confidence intervals (CI) for death from any cause for the subgroup (patients treated with at least three lines of treatment) after start of first line metastatic castration resistant prostate cancer (mCRPC) treatment, adjustment with prognostic factors grouped; step 1: curative treatment, time from start of ADT to CRPC; step 2: lymph node metastases, visceral metastases, laboratory values of PSA, ALP, Hb; step 3: age, ECOG performance status, MDCI, DCI, systemic treatment prior to CRPC, pain.

Line of treatment: line of radium-223 treatment in mCPRC setting; curative treatment: primary treatment with our without curative intent (radiotherapy or radical prostatectomy); ADT: androgen deprivation therapy; CRPC: castration resistant prostate cancer; lymph node metastases: regional lymph node metastases; visceral metastases: visceral metastases including lymph nodes outside the pelvis; PSA: serum prostatic-specific antigen; ALP: serum alkaline phosphatase; Hb: Haemoglobin; ECOG: Eastern Cooperative Oncology Group performance status; MDCI: Medical Drug Comorbidity Index; DCI: Drug Comorbidity Index; systemic treatment prior to CRPC: abiraterone/enzalutamide, docetaxel, the combination of them and any other chemotherapy for prostate cancer; pain: dispensed opioid prescription within 60 days before start of radium-223.
